# NaCl Potentiates Human Fibrocyte Differentiation

**DOI:** 10.1371/journal.pone.0045674

**Published:** 2012-09-18

**Authors:** Nehemiah Cox, Darrell Pilling, Richard H. Gomer

**Affiliations:** Department of Biology, Texas A&M University, College Station, Texas, United States of America; University Medical Center Freiburg, Germany

## Abstract

Excessive NaCl intake is associated with a variety of fibrosing diseases such as renal and cardiac fibrosis. This association has been attributed to increased blood pressure as the result of high NaCl intake. However, studies in patients with high NaCl intake and fibrosis reveal a connection between NaCl intake and fibrosis that is independent of blood pressure. We find that increasing the extracellular concentration of NaCl to levels that may occur in human blood after high-salt intake can potentiate, in serum-free culture conditions, the differentiation of freshly-isolated human monocytes into fibroblast-like cells called fibrocytes. NaCl affects the monocytes directly during their adhesion. Potassium chloride and sodium nitrate also potentiate fibrocyte differentiation. The plasma protein Serum Amyloid P (SAP) inhibits fibrocyte differentiation. High levels of extracellular NaCl change the SAP Hill coefficient from 1.7 to 0.8, and cause a four-fold increase in the concentration of SAP needed to inhibit fibrocyte differentiation by 95%. Together, our data suggest that NaCl potentiates fibrocyte differentiation. NaCl-increased fibrocyte differentiation may thus contribute to NaCl-increased renal and cardiac fibrosis.

## Introduction

Fibrosing diseases such as pulmonary fibrosis, congestive heart disease, and renal fibrosis involve the formation of unwanted scar tissue in internal organs [1], [2]. The scar tissue in fibrosis leads to organ malfunction and subsequent organ failure [1]. Fibrosis is associated with approximately 45% of deaths in the U.S [1]. Fibrosis involves infiltration of blood leukocytes into the affected organs, activation and/or appearance of fibroblast-like cells, tissue remodeling, and deposition of extracellular matrix proteins such as collagen [3]–[8]. Fibrosis can be caused by factors such as environmental toxins, or aberrant healing events [6].

Fibrocytes are CD45^+^, collagen I^+^ fibroblast-like cells that have been implicated in scar tissue formation and fibrosis [3], [5], [6], [8], [9]. These cells share similarities with both blood leukocytes and tissue resident cells [7], [9]. Fibrocytes, depending on the environmental cues, can express extracellular proteases, or matrix proteins such as collagen [6], [10]. Fibrocytes differentiate from CD14^+^ monocytes [8]. Following their recruitment to a specific tissue, monocytes can differentiate into macrophages, dendritic cells, or fibrocytes [6], [11], [12]. Serum Amyloid P (SAP), a pentameric protein from the pentraxin family of proteins, interacts with Fc receptors on monocytes to inhibit fibrocyte differentiation [13]–[16].

Monocytes leave the bone marrow and travel through the blood vessels until they are recruited into a specific tissue in response to chemokines. Afterward, they mature and differentiate under the influence of signaling molecules such as M-CSF, GM-CSF, IL-4, IL-13, and TGF-β1 [17]–[20]. One component of monocyte activation and differentiation is a group of receptors belonging to the integrin family of proteins [21], [22]. Integrins are composed of α and β subunits [23]. These proteins aid in monocyte adhesion to components of extracellular matrices and are central to inflammation, immunity, and homeostasis [24]. The adhesive properties of integrins contribute to different patterns of monocyte differentiation under various conditions [21], [22], [25].

Much remains to be understood about the mechanisms which trigger fibrosis and fibrocyte differentiation. Congestive heart disease and renal fibrosis have previously been linked to high NaCl intake in both humans and various animal models, but the exact mechanism underlying this connection is unknown [2], [26]–[30]. Sodium and chloride ions contribute to the maintenance of electrical gradients across the membrane of many cells. In addition, sodium and chloride ions are used to absorb nutrients such as amino acids in the intestine, and regulate blood pressure and volume [26], [31], [32]. The latter function of sodium and chloride ions has been the focus of many studies, since abnormalities in blood pressure have been associated with stroke, cardiac fibrosis, and renal fibrosis [27].

It is generally accepted that there is a direct correlation between high salt intake and high blood pressure which in turn increases the chances of heart disease, stroke and renal failure [27]. Until recently, it was believed that high blood pressure is the main contributor to heart disease and renal failure. However, animal and clinical studies have shown the deleterious effects (i.e. stroke, cardiac and renal fibrosis) of salt in the absence of increased blood pressure [26], [29], [33], [34]. For instance, Wistar or Wistar-Kyoto rats exposed to salt overload develop cardiac, vascular, and renal fibrosis without exhibiting a significant increase in blood pressure [28], [29]. In addition, a connection between salt intake and fibrosis that is independent of high blood pressure has been previously established [26], [35], [36].

**Figure 1:**
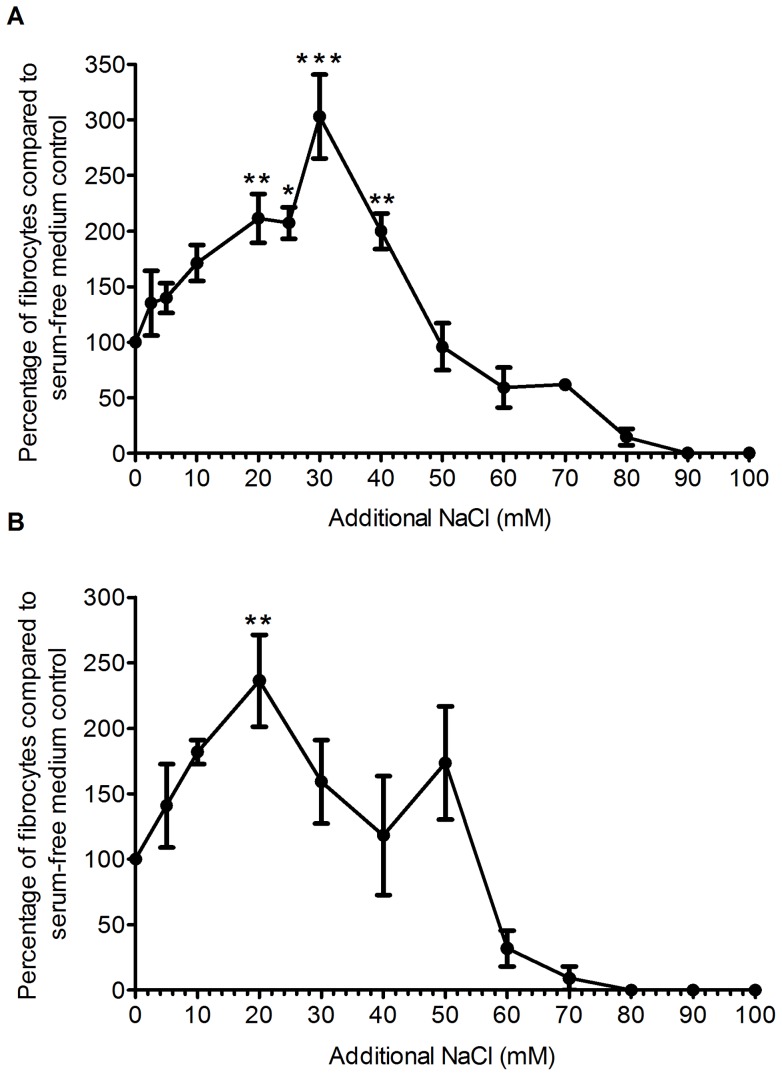
Addition of NaCl to Fibrolife or RPMI based-medium increases fibrocyte differentiation. Human PBMCs were cultured for 5 days in serum-free medium in the presence of the indicated concentrations of additional NaCl in either Fibrolife (A) or RPMI (B) medium. Results are expressed as mean ± SEM, n = 8. * indicates statistical significance with p<0.05, ** indicates P<0.01, and *** indicates p<0.001 by ANOVA compared to the no additional NaCl control.

**Figure 2:**
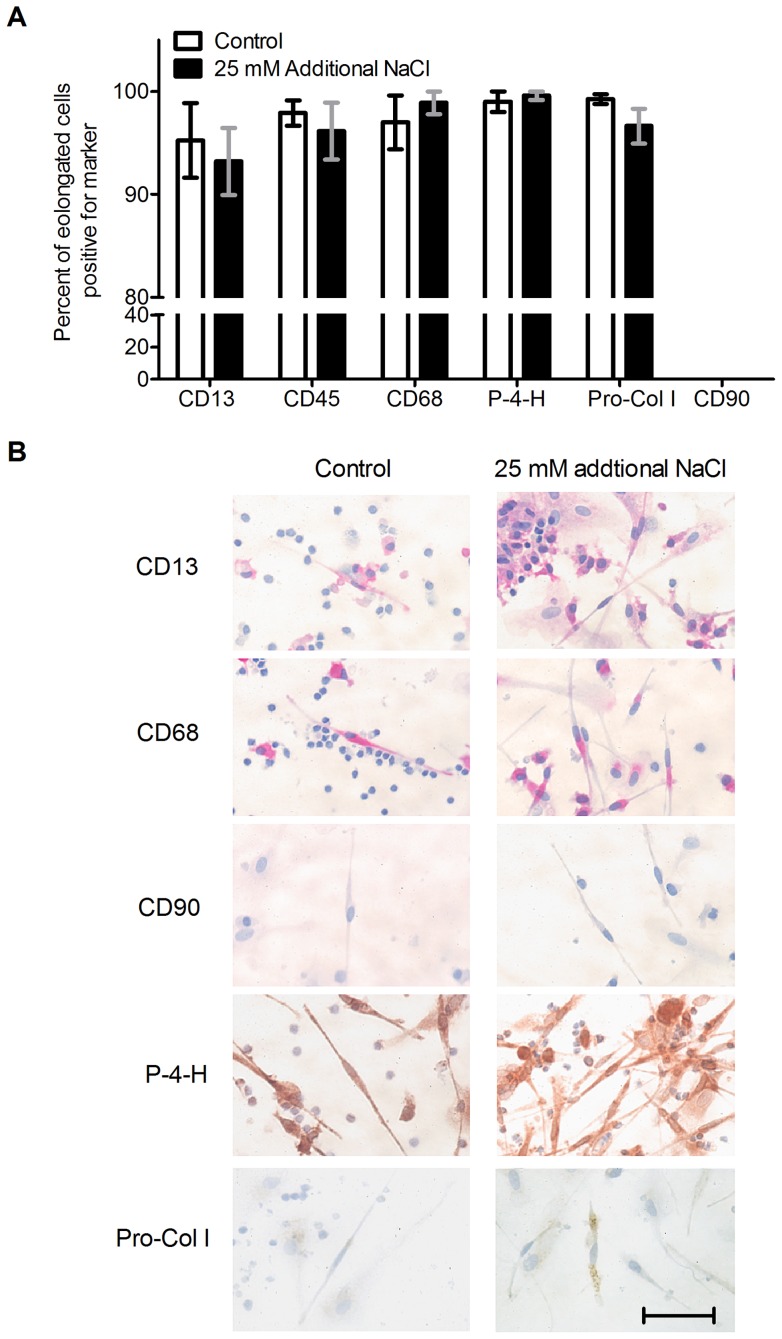
The elongated cells are fibrocytes. PBMCs were cultured in Fibrolife medium for five days in the presence or absence of 25 mM additional NaCl. Cells were fixed and stained for the indicated markers. (A) The elongated spindle-shaped cells in the wells stained positive for CD13, CD45, CD68, Prolyl 4-hydroxylase (P-4-H), Pro-Collagen I (Pro-Col I) and had no apparent staining for the fibroblast marker CD90. Results are expressed as mean ± SEM, n = 3. (B) Cells were stained with the indicated antibodies. Positive staining was identified by red for alkaline phosphatase staining and brown for peroxidase staining. Cells were counterstained blue with hematoxylin to identify nuclei. Size bar is 50 µm.

A variety of studies indicate that different ions can modulate immune cell function. For instance, potassium channels and potassium transport affect the integrin-dependent activation of the monocytic-derived cell line, THP-1 [37]. Altering cation transport in murine erythroleukemia cells can induce differentiation [38]. This, combined with the increased instances of fibrosing diseases in patients with high dietary salt intake, caused us to investigate the role of NaCl in the activation of monocytes and their subsequent differentiation into fibrocytes. Our results suggest that high concentrations of NaCl potentiate fibrocyte formation.

**Figure 3:**
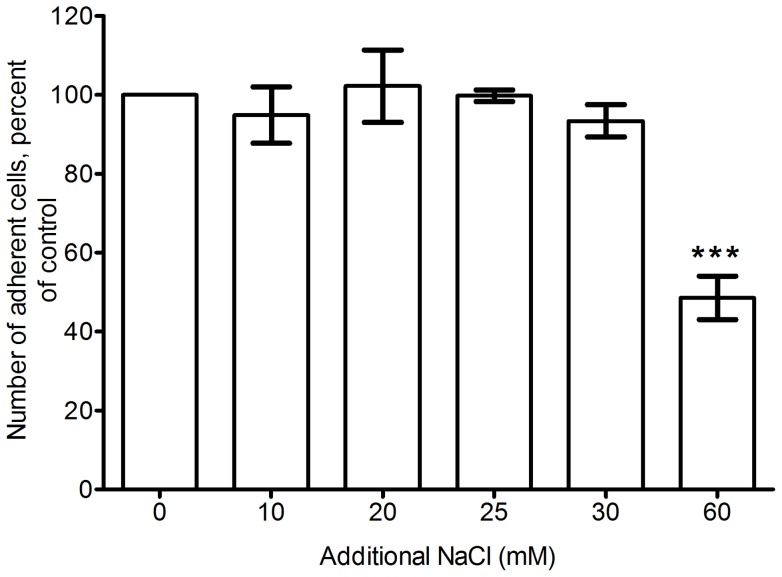
The effect of NaCl on adherent cells after 5 days. PBMCs were incubated with the indicated concentrations of added NaCl, and the number of adherent cells was counted after 5 days. Counts were then normalized to the control with no added NaCl (0). Values are mean ± SEM, n = 3. *** indicates statistical significance by ANOVA compared to the control (p<0.001).

## Results

### Additional NaCl can potentiate fibrocyte formation without influencing cell viability

Since fibrocytes are implicated in fibrosing diseases, and NaCl may promote fibrosis, we examined the hypothesis that NaCl might affect fibrocyte differentiation. Using PBMCs from a variety of donors, we observed 600 to 2800 fibrocytes per 10^5^ PBMCs in both Fibrolife and RPMI 1640 medium. For all donors, additional NaCl significantly potentiated fibrocyte differentiation, when compared to the control with no added NaCl (Figure 1A and B). In Fibrolife medium, NaCl caused a maximum potentiation of fibrocyte differentiation at 30 mM additional NaCl. The [Na^+^] and [Cl^−^] concentrations in Fibrolife medium are 126 mM and 119 mM, respectively. At 30 mM additional NaCl, both the sodium and the chloride concentration of Fibrolife medium ([Na^+^]  = 156 mM, [Cl^−^] = 144 mM) are in excess when compared to human blood ([Na^+^] = 137 mM, [Cl^+^] = 102 mM) [39], [40]. The [Na^+^] and [Cl^−^] concentrations in RPMI 1640 medium are 134 mM and 107 mM, respectively. In RPMI 1640 medium, NaCl caused a maximum potentiation of fibrocyte differentiation at 20 mM additional NaCl, making [Na^+^]  = 154 mM, and [Cl^−^] = 127 mM, again in excess when compared to human blood.

**Figure 4:**
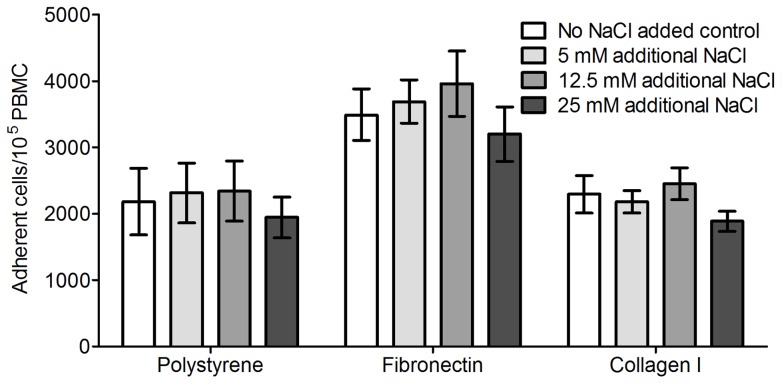
NaCl does not influence the adhesion of PBMCs to plastic, plasma fibronectin or collagen I. PBMCs were incubated on uncoated polystyrene plates, fibronectin coated plates, or bovine collagen I coated plates for one hour in the indicated concentrations of additional NaCl. The non-adhered cells were then rinsed off and the number of adherent cells were counted. Values are mean ± SEM, n = 4.

To verify that the NaCl-induced spindle-shaped cells are fibrocytes, we stained PBMCs after 5 days of incubation with or without 25 mM additional NaCl in Fibrolife for fibrocyte markers. In the presence or absence of added NaCl, the elongated cells were positive for the fibrocyte markers CD13, CD45, CD68, pro-collagen I, and prolyl 4-hydroxylase, and showed no observable staining for the fibroblast marker CD90, indicating that the elongated cells are fibrocytes, and that NaCl thus is increasing the number of fibrocytes (Figure 2).

**Figure 5:**
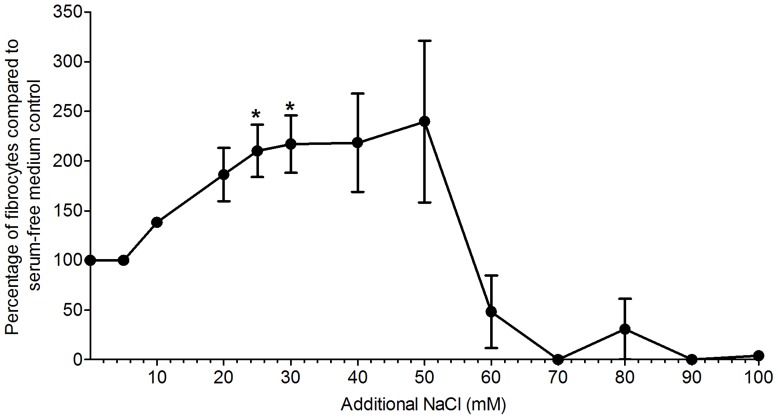
NaCl directly potentiates the differentiation of monocytes into fibrocytes. CD14^+^ monocytes were incubated with the indicated additional concentrations of NaCl. After 5 days, fibrocytes were counted. Values are mean ± SEM, n = 3. * indicates p<0.05 compared to control (ANOVA). The absence of error bars indicate that error was smaller than the plot symbol.

An increase in the total number of adherent cells in our assay plates could account for the increased fibrocyte count. To address this possibility, we counted the number of adherent cells after 5 days of incubation with additional NaCl. At 30 mM additional NaCl and below, we observed no significant change in the number of adherent cells as the result of additional NaCl (Figure 3). The addition of 60 mM additional NaCl did however decrease the number of adherent cells, indicating reduced cell viability.

**Figure 6:**
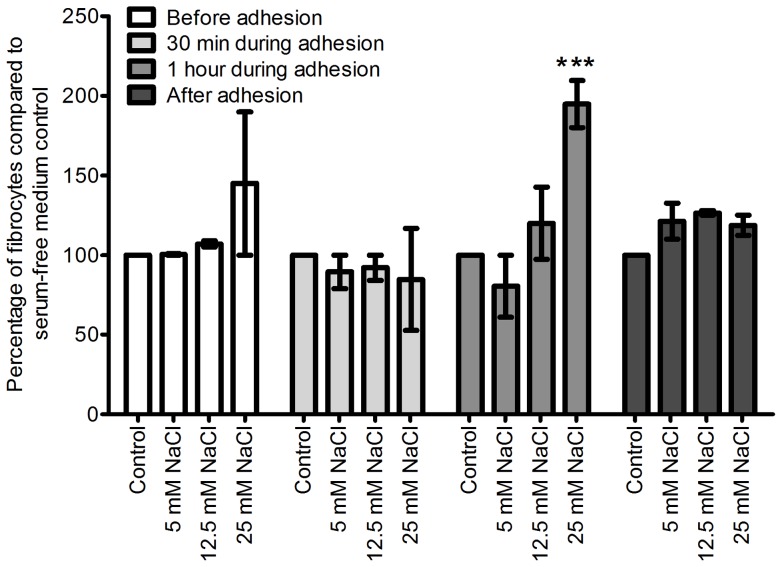
The presence of additional NaCl during PBMC adhesion increases fibrocyte differentiation. PBMCs were incubated with additional NaCl, before, during, before, and after their adhesion to tissue culture treated plates. Values are mean ± SEM, n = 6. *** indicates p<0.001 compared to control (t-test). The absence of an error bar indicates that the error was smaller than the line thickness.

### 25 mM additional NaCl does not influence PBMC adhesion

Fibrocytes differentiate from CD14^+^ monocytes [6], [8]. Monocytes are activated in response to adhesion to extracellular matrix proteins as well as tissue culture plates [11], [12]. To understand the mechanism behind the potentiation of fibrocyte formation by NaCl, we investigated the influence of NaCl on the initial adhesion of PBMCs to plates, since such changes could influence PBMC activation and therefore differentiation. PBMC adhesion to collagen I-coated, fibronectin-coated, or un-coated tissue culture plates was not influenced by increasing NaCl concentrations (Figure 4). However, as previously observed, more PBMCs adhered to fibronectin-coated plates than un-coated plates ([41] and Figure 4). These results suggest that the observed increase in fibrocyte differentiation is not caused by changes in the ability of PBMCs to initially adhere to extracellular matrix proteins or tissue culture plates.

**Figure 7:**
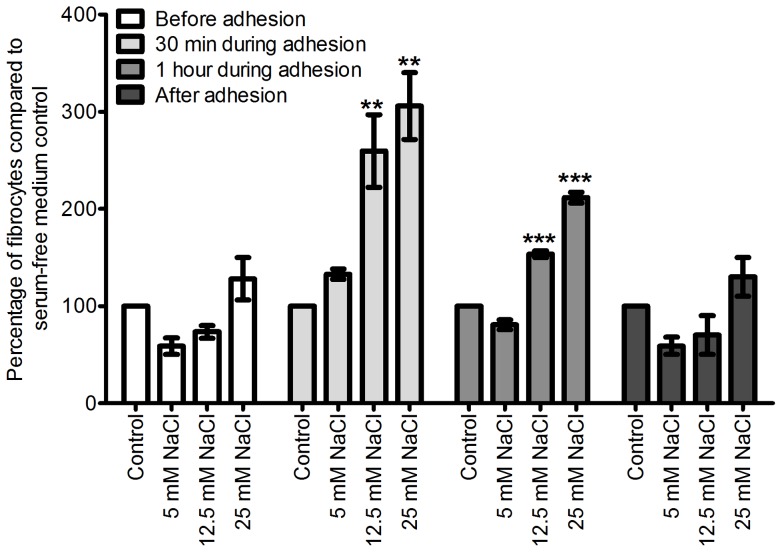
The presence of additional NaCl during CD14^+^ monocyte adhesion increases fibrocyte formation. Isolated monocytes were incubated with additional NaCl before, during, and after their adhesion to tissue culture treated plates. Values are mean ± SEM, n = 4, ** indicates p<0.01, and *** indicates p<0.001 compared to control (ANOVA).

### Monocyte to fibrocyte differentiation is potentiated by additional NaCl

The NaCl-induced increase in fibrocyte formation from PBMCs could be due to either secretion of a fibrocyte stimulating factor by non-monocytic cells, or via direct effect of NaCl on monocytes. To distinguish between these two possibilities, we isolated CD14^+^ cells and examined their differentiation into fibrocytes in the presence of increasing NaCl concentrations. We observed 1400 to 9200 fibrocytes per 10^5^ monocytes from 3 different donors. When fibrocyte counts were compared to the no NaCl added control, fibrocyte differentiation was significantly increased by 20 mM to 30 mM additional NaCl (Figure 5). This suggests that monocytes are directly influenced by the additional NaCl, and not through a secreted factor from CD14-negative cells.

**Figure 8:**
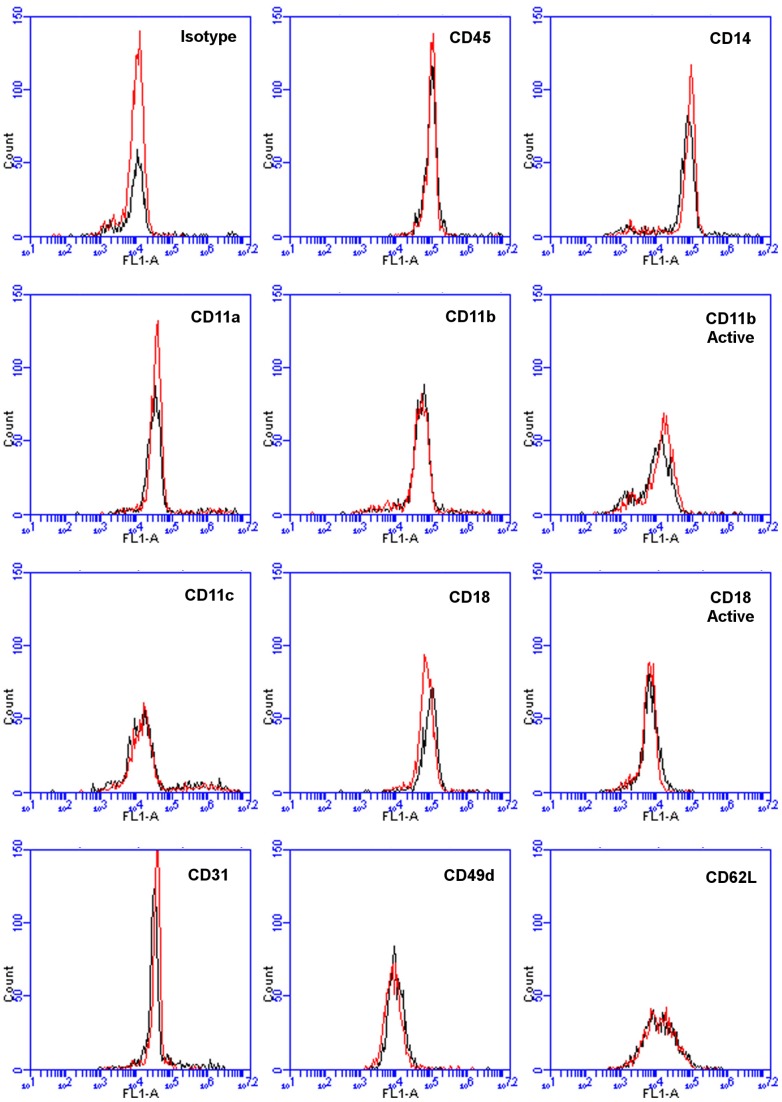
Incubation of PBMCs with additional NaCl does not influence the cell-surface levels of several adhesion molecules. PBMCs were incubated in the presence or absence of 25 mM additional NaCl for 1 hour at 37°C and then stained for the indicated adhesion molecules. Monocytes were identified by their forward and side scatter characteristics and the expression of CD14. Flow cytometry plots are representative results from three separate donors. Histograms represent fluorescence intensity of the indicated marker in control cells (Black line) and NaCl treated Cells (Red line).

### PBMCs and monocytes are influenced during their adhesion by additional NaCl

PBMCs are activated by their adhesion to extracellular matrix proteins and tissue culture plates [42]. To elucidate the mechanism behind the NaCl-induced increase in fibrocyte formation, we incubated PBMCs with additional NaCl before, during, and after their adhesion (Figure 6). We observed that incubation of PBMCs with additional NaCl for one hour during their adhesion potentiated fibrocyte formation (Figure 6). Incubation of PBMCs with additional NaCl before or after their adhesion, or for 30 minutes during their adhesion, had no significant effect on fibrocyte formation (Figure 6). These data suggest that NaCl influences fibrocyte precursors during adhesion.

**Figure 9:**
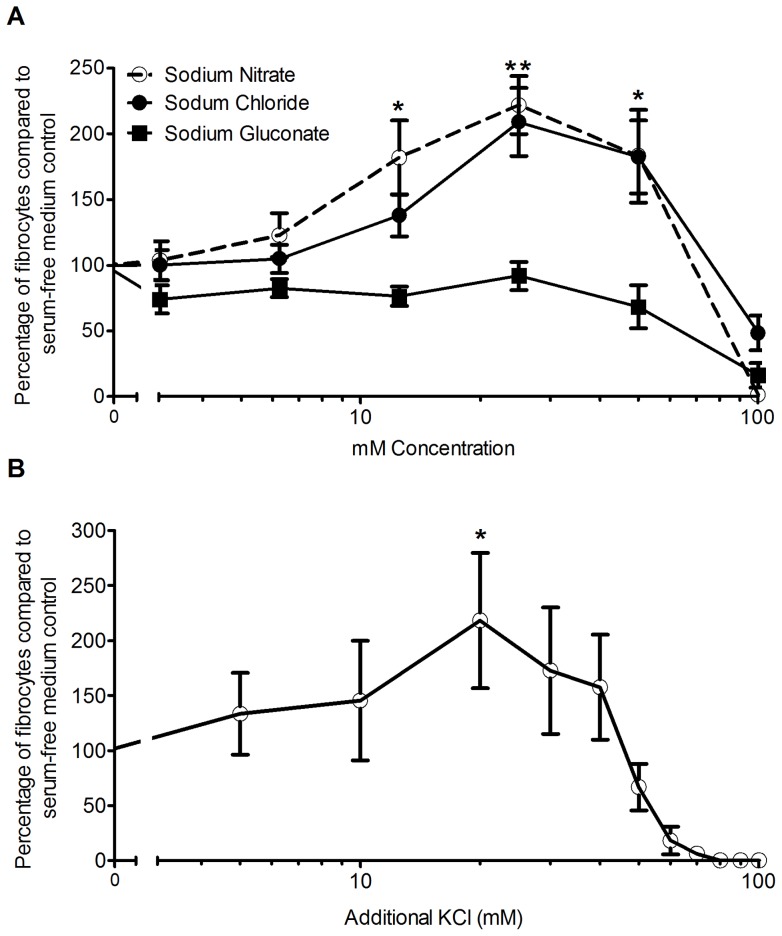
Sodium nitrate and potassium chloride potentiate fibrocyte differentiation. PBMCs were cultured with the indicated concentrations of additional (A) sodium chloride, sodium nitrate, or sodium gluconate, or (B) potassium chloride. Values are mean ± SEM, n = 6. * indicates p<0.05, and ** indicated p<0.01 compared to control by ANOVA.

CD14^+^ monocytes were also incubated with additional NaCl before, during, and after adhesion. Similar to the results with PBMCs, incubation of monocytes with additional NaCl during their adhesion resulted in increased fibrocyte formation (Figure 7). A 30-minute incubation of monocytes with 25 mM additional NaCl was sufficient for this potentiation.

**Figure 10:**
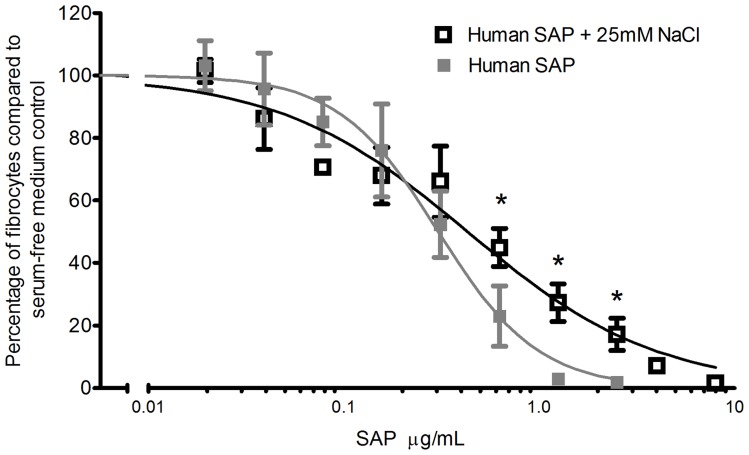
NaCl interferes with the ability of hSAP to inhibit fibrocyte differentiation. PBMCs were incubated with or without additional 25 mM NaCl with the indicated concentrations of human SAP (hSAP). After 5 days, fibrocytes were counted. Values are mean ± SEM, n = 5. * indicates p<0.05 compared to SAP alone control (dashed line) by ANOVA. The absence of error bars indicate that error was smaller than the plot symbol. Lines are sigmoidal dose-response curves with variable Hill coefficient fit to the data.

Since additional NaCl potentiates fibrocyte differentiation by influencing monocytes during their initial adhesion to the plate, we examined the effects of 25 mM additional NaCl on different adhesion molecules. We incubated PBMCs in the presence or absence of 25 mM additional NaCl for 1 hour at 37°C. Following incubation, the cells were stained for CD11a, CD11b, CD11c, CD11b activation epitope, CD18, CD18 activation epitope, CD31, CD49d, and CD62L. The stained cells were subjected to flow cytometry and the CD14^+^ monocytes were investigated for any change in the mentioned adhesion molecules. We observed no change in the studied adhesion molecules as the result of additional NaCl (Figure 8).

### Sodium nitrate but not sodium gluconate potentiates fibrocyte differentiation

Sodium nitrate and sodium gluconate are two classical sodium chloride substitutes used to probe chloride transport [43]. Nitrate can bind and cross chloride channels [43]–[46]. Gluconate only mimics the electrical charge of chloride while being unable to cross the majority of chloride channels [43]–[47]. Sodium nitrate but not sodium gluconate potentiated fibrocyte differentiation (Figure 9A). The response of PBMCs to sodium nitrate was similar to that of NaCl. This suggests that chloride transport across the membrane is a requirement for potentiation of fibrocyte differentiation by additional NaCl. In addition to sodium nitrate and sodium gluconate, we investigated other compounds such as potassium chloride, rubidium chloride, cesium chloride, choline chloride, tetraethyl ammonium chloride, and monosodium glutamate. Adding 25 mM potassium chloride potentiated fibrocyte differentiation (Figure 9B). Monosodium glutamate had no effect of fibrocyte differentiation but was toxic when added in excess of 50 mM. Choline chloride and rubidium chloride were toxic at concentrations higher than 4 mM, while cesium chloride and tetraethyl ammonium chloride were toxic at 2 mM (data not shown).

### NaCl interferes with the ability of Serum Amyloid P to inhibit fibrocyte differentiation

Human Serum Amyloid P (hSAP) is a pentameric protein secreted from the liver that inhibits fibrocyte differentiation [13], [48], [49]. The addition of 25 mM NaCl to the medium changed the Hill coefficient for the inhibition of fibrocyte differentiation by hSAP from 1.7±0.1 to 0.7±0.1 (mean ± SEM, n = 5, p<0.05 by t-test) (Figure 10). In addition, adding 25 mM to the medium significantly inhibited the ability of high concentrations of hSAP to inhibit fibrocyte differentiation (Figure 10). The data indicate that NaCl, in addition to potentiating fibrocyte differentiation, influences how hSAP inhibits fibrocyte differentiation.

## Discussion

Monocyte-derived fibrocytes are found in fibrotic lesions, skin wounds, and tumors [3], [5]–[7], [50]–[53]. We have shown here that the differentiation of human monocytes into fibrocytes in serum-free culture is potentiated by NaCl. This increase appears to be a direct effect of NaCl on monocytes. In patients with long-term elevated salt intake, there is a 5% increase in serum NaCl, raising [Na^+^] from 137 mM to 144 mM [40]. In addition, food intake increases the blood sodium chloride level for ∼4 hours [54]. We observed that 155 mM [Na^+^] and 130 [Cl−] potentiates fibrocyte differentiation, and we hypothesize that this [Na^+^] and [Cl−] concentration could occur in the serum or tissues of some individuals, at least for a few hours after eating a high-salt meal. However short, this transient increase could be sufficient to increase fibrocyte differentiation.

In animal models and humans, increasing the extracellular osmolarity suppresses neutrophil functions such as cellular cytotoxicity and migration [55], [56]. In addition, when tested on PBMCs in vitro, high salt increases the secretion of the pro-inflammatory cytokines IL-8 and TNF-α [57]–[59]. These studies indicate that extracellular osmolarity can affect cells of the immune system. The increase in fibrocyte differentiation in response to increased NaCl could thus be due to increased osmolarity. However, we observed that adding up to 50 mM sodium gluconate or monosodium glutamate to the medium, which would also increase osmolarity, did not affect fibrocyte differentiation. Alternatively, an increase in cell proliferation as the result of additional NaCl could explain the increase in fibrocyte count. However, previous studies on the proliferation of fibrocytes and fibrocyte precursors revealed no appreciable proliferation [60]. Thus, the observed increase in fibrocyte differentiation is probably not related to the medium osmolarity or cell proliferation in our in vitro assay and most likely involves a direct effect of NaCl on monocytes.

When monocytes are activated following their recruitment into the tissue and adhesion to extracellular matrices, they undergo changes in gene expression and receptor levels [42], [61], [62]. Following adhesion, monocytes are activated through a series of intracellular signaling events. We found that NaCl was most effective at increasing fibrocyte differentiation during monocyte adhesion. This was not accompanied by any changes in the expression of adhesion molecules CD11a, CD11b, CD11c, CD18, CD31, CD49d, and CD62L. In agreement with this, the additional NaCl did not influence the adhesive properties of monocytes to plasma fibronectin, collagen I, or un-coated tissue culture plates. Membrane potential and ion transport affect the activation of the monocyte-derived cell line THP-1 [37]. It is thus possible that the effect of NaCl on fibrocyte differentiation is largely due to the increased NaCl affecting membrane potential and ion transport during the adhesion stage of monocyte activation.

Ion channels, especially chloride channels, appear to affect cellular activation and differentiation. In osteoblasts, the chloride channel ClC-3 regulates osteo-differentiation [63]. The development and differentiation of keratinocytes is regulated in part by calcium-gated chloride channels [64]. We observed that sodium nitrate but not sodium gluconate potentiated fibrocyte differentiation. Nitrate can pass through chloride channels, while gluconate cannot [43], [46]. Combined with the observation that KCl potentates fibrocyte differentiation, this suggests that chloride ions might play a role in the NaCl-induced potentiation of fibrocyte differentiation.

Monocyte activation and the subsequent differentiation into fibrocyte is influenced by variety factors such as SAP, M-CSF, GM-CSF, IL-4, IL-13 and, TGF-β1 [8], [13], [60]. These regulators of the immune system each use distinct and sometimes overlapping signaling transduction pathways making it difficult to identify the exact pathway responsible for the NaCl-induced increase in fibrocyte formation.

Excessive intake of NaCl has been associated with high blood pressure, stroke, heart disease, and kidney disease [26], [27], [65], [66]. The correlation between high salt intake and these diseases has generally been attributed to high blood pressure [27]. However, studies in rats have questioned this correlation [28], [29]. In addition, two independent studies on cohorts of patients with high salt intake and fibrosis showed a connection between these two factors that was independent of high blood pressure [35], [36]. Here we have shown that fibrocyte differentiation can be potentiated by increasing extracellular NaCl levels in vitro. An intriguing possibility is that the link between high sodium chloride intake and fibrosis involves NaCl potentiating fibrocyte differentiation.

In addition to participating in fibrosis, fibrocytes participate in wound healing [6], [7], [67]. Our observations suggest that having ∼155 mM [Na^+^] and ∼ 130 mM [Cl^−^] in wound dressings could not only potentiate fibrocyte differentiation but also influence the ability of hSAP to inhibit fibrocyte differentiation, and thus potentiate wound healing.

## Materials and Methods

### PBMC and monocyte isolation, cell culture, and fibrocyte differentiation assay

Human blood was collected into heparin tubes (BD Bioscience, San Jose, CA) from adult volunteers who gave written consent and with specific approval from the Texas A&M University human subjects Institutional Review Board. Peripheral blood mononuclear cells (PBMCs) were isolated from the blood by Ficoll-Paque Plus (GE Healthcare Biosciences, Piscataway, NJ) and cultured in Fibrolife (LifeLine Cell Technology, Walkersville, MD) medium or where indicated in RPMI 1640 (SigmaAldrich, St. Louis, MO) as described previously [68]. CD14^+^ monocytes were isolated by negative selection using magnetic Dynabeads (Dynal Biotech, Milwaukee, WI ) as described previously [13]. CD14^+^ monocytes were plated in 96-well plates with 5×10^4^ cells in 200 µl per well. After 5 days, the plates were air dried, fixed with methanol and stained using a Hema 3 staining Kit (Thermo Fisher Scientific, Milwaukee, WI) [68]. Fibrocytes were identified and counted based on their elongated spindle-shaped morphology in five different 900 µm-diameter fields of view per well.

### Salt Solutions and Serum Amyloid P purification

One molar solutions of sodium chloride, sodium gluconate, sodium nitrate, and other salts were prepared in Fibrolife (LifeLine Cell Technology) basal medium or RPMI-1640 (SigmaAldrich) where indicated. The 1 M solutions were then filter sterilized using Acrodisc 0.2 µm syringe filters (Pall, Port Washington, NY). Prepared solutions were then added to the wells at the indicated concentrations. Human Serum Amyloid P was purified from human serum as described previously [49].

### Immunohistochemistry

PBMCs were cultured for 5 days on eight-well glass microscope slides (Thermo Fisher) as described previously [9]. The cells were then air dried before fixation in acetone for 10 minutes. The cells were stained for CD13, CD45, CD68, Pro-collagen I (Developmental Studies Hybridoma Bank, University of Iowa), CD90, and Prolyl 4-hydroxylase as described previously [9].

### Adhesion Assay

96-well polystyrene plates (BD Biosciences) were coated with 15 ug/mL of bovine plasma fibronectin (SigmaAldrich) or bovine collagen I (SigmaAldrich) for 1 hour at 37°C. The plate was then washed 3 times with PBS and then blocked with 4% BSA (SigmaAldrich, cat # A3059) in PBS for 1 hour at 37°C. Afterward, the plate was washed with serum-free medium, and peripheral blood mononuclear cells were incubated on the plate for 1 hour at 37°C. The plates were then washed 3 times with PBS and adherent cells were counted in five 900 µm-diameter fields of view per well.

### PBMC/monocyte pulse experiment

PBMCs or monocytes were incubated with additional concentrations of NaCl for the indicated times at 37°C to determine the effect of additional of NaCl on fibrocyte differentiation during adhesion. Then, approximately 95% of the medium was gently removed, so as not to disturb the cells, and replaced with fresh medium. The cells were left to differentiate for 5 days. To determine the effect of NaCl on adherent cells, PBMCs or monocytes were incubated in 96-well plates for 1 hour at 37°C, and the medium was then replaced with medium containing additional NaCl. The cells were left for 1 hour at 37°C, and subsequently the medium was replaced with fresh medium with no additional NaCl. These cells were then left to differentiate for 5 days. To elucidate the effect of NaCl on PBMCs or monocytes before their adhesion to the plate, cells were placed in BSA-coated eppendorf tubes for 1 hour in medium with additional NaCl at 4°C. The cells were then collected by centrifugation for 5 minutes at 300 x g. The pelleted cells were resuspended in fresh medium and incubated in a 96-well plate for 5 days at 37°C to differentiate.

### Adhesion molecules and flow cytometry

96-well polystyrene plates (BD Biosciences) were coated with 20 µg/mL of plasma fibronectin (SigmaAldrich) overnight at 4°C. The following day the coated wells were washed 3 x with PBS and blocked with 2% BSA in PBS. After blocking, the wells were washed 3x with PBS and then the PBMCs were plated with 2×10^5^ cells per well in the presence or absence of 25 mM additional NaCl for 1 hour at 37°C. After the incubation, the medium was replaced with PBS/10 mM EDTA and the plate was left at 4°C for 15 minutes to detach the cells from the wells. The detached cells were then pelleted by centrifugation at 300 x g for 5 minutes and resuspended in 2% BSA in PBS. Following centrifugation, the cells were stained for CD11a (BioLegend, San Diego, CA), CD11b (BioLegend), CD11c (BioLegend), CD11b activation epitope (BioLegend), CD18 (BioLegend), CD18 activation epitope (Abd Serotec), CD31 (BioLegend), CD45 (BioLegend), CD49d (BioLegend) and, CD62L (BioLegend) as described previously [9].

### Statistical analysis

Data was analyzed by ANOVA (with Dunnett's post test) or t-test when appropriate using Prism software (GraphPad software, San Diego, CA). Normality was tested using Shapiro-Wilk and D'Agostino-Pearson omnibus tests.

## References

[1] Wynn TA (2004) Fibrotic disease and the T(H)1/T(H)2 paradigm. Nat Rev Immunol 4: 583–594. 15286725 10.1038/nri1412 PMC2702150

[2] Sakai N, Wada T, Yokoyama H, Lipp M, Ueha S, et al (2006) Secondary lymphoid tissue chemokine (SLC/CCL21)/CCR7 signaling regulates fibrocytes in renal fibrosis. Proc Natl Acad Sci U S A 103: 14098–14103. 16966615 10.1073/pnas.0511200103 PMC1599918

[3] Moeller A, Gilpin SE, Ask K, Cox G, Cook D, et al (2009) Circulating fibrocytes are an indicator of poor prognosis in idiopathic pulmonary fibrosis. Am J Respir Crit Care Med 179: 588–594. 19151190 10.1164/rccm.200810-1534OC

[4] McAnulty RJ (2007) Fibroblasts and myofibroblasts: their source, function and role in disease. Int J Biochem Cell Biol 39: 666–671. 17196874 10.1016/j.biocel.2006.11.005

[5] Quan TE, Cowper SE, Bucala R (2006) The role of circulating fibrocytes in fibrosis. Curr Rheumatol Rep 8: 145–150. 16569374 10.1007/s11926-006-0055-x

[6] Reilkoff RA, Bucala R, Herzog EL (2011) Fibrocytes: emerging effector cells in chronic inflammation. Nat Rev Immunol 11: 427–435. 21597472 10.1038/nri2990 PMC3599774

[7] Bucala R, Spiegel LA, Chesney J, Hogan M, Cerami A (1994) Circulating fibrocytes define a new leukocyte subpopulation that mediates tissue repair. Mol Med 1: 71–81. 8790603 PMC2229929

[8] Abe R, Donnelly SC, Peng T, Bucala R, Metz CN (2001) Peripheral blood fibrocytes: differentiation pathway and migration to wound sites. J Immunol 166: 7556–7562. 11390511 10.4049/jimmunol.166.12.7556

[9] Pilling D, Fan T, Huang D, Kaul B, Gomer RH (2009) Identification of markers that distinguish monocyte-derived fibrocytes from monocytes, macrophages, and fibroblasts. PloS one 4: e7475. 19834619 10.1371/journal.pone.0007475 PMC2759556

[10] Strieter RM, Keeley EC, Burdick MD, Mehrad B (2009) The role of circulating mesenchymal progenitor cells, fibrocytes, in promoting pulmonary fibrosis. Trans Am Clin Climatol Assoc 120: 49–59. 19768162 PMC2744511

[11] Gordon S, Taylor PR (2005) Monocyte and macrophage heterogeneity. Nat Rev Immunol 5: 953–964. 16322748 10.1038/nri1733

[12] Auffray C, Sieweke MH, Geissmann F (2009) Blood monocytes: development, heterogeneity, and relationship with dendritic cells. Annu Rev Immunol 27: 669–692. 19132917 10.1146/annurev.immunol.021908.132557

[13] Pilling D, Buckley CD, Salmon M, Gomer RH (2003) Inhibition of fibrocyte differentiation by serum amyloid P. J Immunol. 171: 5537–5546. 10.4049/jimmunol.171.10.5537 PMC4482350 14607961

[14] Hutchinson WL, Hohenester E, Pepys MB (2000) Human serum amyloid P component is a single uncomplexed pentamer in whole serum. Mol Med 6: 482–493. 10972085 PMC1949963

[15] Castaño AP, Lin SL, Surowy T, Nowlin BT, Turlapati SA, et al (2009) Serum amyloid P inhibits fibrosis through Fc gamma R-dependent monocyte-macrophage regulation in vivo. Sci Transl Med 1: 5ra13. 10.1126/scitranslmed.3000111 PMC2852889 20368175

[16] Pilling D, Tucker NM, Gomer RH (2006) Aggregated IgG inhibits the differentiation of human fibrocytes. J Leukoc Biol 79: 1242–1251. 16543402 10.1189/jlb.0805456 PMC4482138

[17] Hamilton JA, Stanley ER, Burgess AW, Shadduck RK (1980) Stimulation of macrophage plasminogen activator activity by colony-stimulating factors. J Cell Physiol 103: 435–445. 6967488 10.1002/jcp.1041030309

[18] Mosser DM, Edwards JP (2008) Exploring the full spectrum of macrophage activation. Nat Rev Immunol 8: 958–969. 19029990 10.1038/nri2448 PMC2724991

[19] Wynn TA, Ramalingam TR (2012) Mechanisms of fibrosis: therapeutic translation for fibrotic disease. Nat Med 18: 1028–1040. 22772564 10.1038/nm.2807 PMC3405917

[20] Hamilton JA (2008) Colony-stimulating factors in inflammation and autoimmunity. Nat Rev Immunol 8: 533–544. 18551128 10.1038/nri2356

[21] Shi C, Simon DI (2006) Integrin signals, transcription factors, and monocyte differentiation. Trends Cardiovasc Med 16: 146–152. 16781947 10.1016/j.tcm.2006.03.002

[22] Shi C, Zhang XB, Chen ZP, Sulaiman K, Feinberg MW, et al (2004) Integrin engagement regulates monocyte differentiation through the forkhead transcription factor Foxp1. Journal of Clinical Investigation 114: 408–418. 15286807 10.1172/JCI21100 PMC484980

[23] Hogg N, Patzak I, Willenbrock F (2011) The insider's guide to leukocyte integrin signalling and function. Nat Rev Immunol 11: 416–426. 21597477 10.1038/nri2986

[24] Hynes RO (1992) Integrins: versatility, modulation, and signaling in cell adhesion. Cell 69: 11–25. 1555235 10.1016/0092-8674(92)90115-s

[25] Sudhakaran PR, Radhika A, Jacob SS (2007) Monocyte macrophage differentiation in vitro: Fibronectin-dependent upregulation of certain macrophage-specific activities. Glycoconj J 24: 49–55. 17115276 10.1007/s10719-006-9011-2

[26] He FJ, MacGregor GA (2009) A comprehensive review on salt and health and current experience of worldwide salt reduction programmes. J Hum Hypertens 23: 363–384. 19110538 10.1038/jhh.2008.144

[27] Susic D, Frohlich ED (2012) Salt consumption and cardiovascular, renal, and hypertensive diseases: clinical and mechanistic aspects. Curr Opin Lipidol 23: 11–16. 22123673 10.1097/MOL.0b013e32834d9c52

[28] Yuan BX, Leenen FH (1991) Dietary sodium intake and left ventricular hypertrophy in normotensive rats. Am J Physiol 261: H1397–1401. 1835307 10.1152/ajpheart.1991.261.5.H1397

[29] Yu HC, Burrell LM, Black MJ, Wu LL, Dilley RJ, et al (1998) Salt induces myocardial and renal fibrosis in normotensive and hypertensive rats. Circulation 98: 2621–2628. 9843472 10.1161/01.cir.98.23.2621

[30] Kagiyama S, Matsumura K, Fukuhara M, Sakagami K, Fujii K, et al (2007) Aldosterone-and-salt-induced cardiac fibrosis is independent from angiotensin II type 1a receptor signaling in mice. Hypertens Res 30: 979–989. 18049031 10.1291/hypres.30.979

[31] Munck LK (1995) Chloride dependent amino acid transport in the human small intestine. Gut 36: 215–219. 7883220 10.1136/gut.36.2.215 PMC1382407

[32] Broer S (2008) Amino acid transport across mammalian intestinal and renal epithelia. Physiol Rev 88: 249–286. 18195088 10.1152/physrev.00018.2006

[33] Frohlich ED, Chien Y, Sesoko S, Pegram BL (1993) Relationship between dietary sodium intake, hemodynamics, and cardiac mass in SHR and WKY rats. Am J Physiol 264: R30–34. 8430884 10.1152/ajpregu.1993.264.1.R30

[34] Perry IJ, Beevers DG (1992) Salt intake and stroke: a possible direct effect. J Hum Hypertens 6: 23–25. 1583626

[35] Strazzullo P, D'Elia L, Kandala NB, Cappuccio FP (2009) Salt intake, stroke, and cardiovascular disease: meta-analysis of prospective studies. BMJ 339: b4567. 19934192 10.1136/bmj.b4567 PMC2782060

[36] He FJ, Burnier M, Macgregor GA (2011) Nutrition in cardiovascular disease: salt in hypertension and heart failure. Eur Heart J 32: 3073–3080. 21705359 10.1093/eurheartj/ehr194

[37] Colden-Stanfield M, Gallin EK (1998) Modulation of K+ currents in monocytes by VCAM-1 and E-selectin on activated human endothelium. Am J Physiol 275: C267–277. 9688858 10.1152/ajpcell.1998.275.1.C267

[38] Arcangeli A, Carla M, Del Bene MR, Becchetti A, Wanke E, et al (1993) Polar/apolar compounds induce leukemia cell differentiation by modulating cell-surface potential. Proc Natl Acad Sci U S A 90: 5858–5862. 8516337 10.1073/pnas.90.12.5858 PMC46822

[39] Lindinger MI, Heigenhauser GJ, McKelvie RS, Jones NL (1992) Blood ion regulation during repeated maximal exercise and recovery in humans. Am J Physiol 262: R126–136. 1733331 10.1152/ajpregu.1992.262.1.R126

[40] Farquhar WB, Paul EE, Prettyman AV, Stillabower ME (2005) Blood pressure and hemodynamic responses to an acute sodium load in humans. J Appl Physiol 99: 1545–1551. 15976364 10.1152/japplphysiol.00262.2005

[41] Tobias JW, Bern MM, Netland PA, Zetter BR (1987) Monocyte adhesion to subendothelial components. Blood 69: 1265–1268. 3828535

[42] Springer TA (1994) Traffic signals for lymphocyte recirculation and leukocyte emigration: the multistep paradigm. Cell 76: 301–314. 7507411 10.1016/0092-8674(94)90337-9

[43] Jentsch TJ, Stein V, Weinreich F, Zdebik AA (2002) Molecular structure and physiological function of chloride channels. Physiol Rev 82: 503–568. 11917096 10.1152/physrev.00029.2001

[44] Fahlke C (2001) Ion permeation and selectivity in ClC-type chloride channels. Am J Physiol Renal Physiol 280: F748–757. 11292616 10.1152/ajprenal.2001.280.5.F748

[45] Qu Z, Hartzell HC (2000) Anion permeation in Ca(2+)-activated Cl(−) channels. J Gen Physiol 116: 825–844. 11099350 10.1085/jgp.116.6.825 PMC2231816

[46] Franciolini F, Nonner W (1987) Anion and cation permeability of a chloride channel in rat hippocampal neurons. J Gen Physiol 90: 453–478. 2445901 10.1085/jgp.90.4.453 PMC2228868

[47] Ashton N, Argent BE, Green R (1991) Characteristics of fluid secretion from isolated rat pancreatic ducts stimulated with secretin and bombesin. J Physiol 435: 533–546. 1770448 10.1113/jphysiol.1991.sp018523 PMC1181475

[48] Haudek SB, Xia Y, Huebener P, Lee JM, Carlson S, et al (2006) Bone marrow-derived fibroblast precursors mediate ischemic cardiomyopathy in mice. Proc Natl Acad Sci U S A 103: 18284–18289. 17114286 10.1073/pnas.0608799103 PMC1643845

[49] Pilling D, Roife D, Wang M, Ronkainen SD, Crawford JR, et al (2007) Reduction of bleomycin-induced pulmonary fibrosis by serum amyloid P. J Immunol. 179: 4035–4044. 10.4049/jimmunol.179.6.4035 PMC4482349 17785842

[50] Bellini A, Mattoli S (2007) The role of the fibrocyte, a bone marrow-derived mesenchymal progenitor, in reactive and reparative fibroses. Lab Invest 87: 858–870. 17607298 10.1038/labinvest.3700654

[51] Kisseleva T, Uchinami H, Feirt N, Quintana-Bustamante O, Segovia JC, et al (2006) Bone marrow-derived fibrocytes participate in pathogenesis of liver fibrosis. J Hepatol 45: 429–438. 16846660 10.1016/j.jhep.2006.04.014

[52] Oh MH, Oh SY, Yu J, Myers AC, Leonard WJ, et al (2011) IL-13 induces skin fibrosis in atopic dermatitis by thymic stromal lymphopoietin. J Immunol 186: 7232–7242. 21576506 10.4049/jimmunol.1100504 PMC3399513

[53] Sakai N, Furuichi K, Shinozaki Y, Yamauchi H, Toyama T, et al (2010) Fibrocytes are involved in the pathogenesis of human chronic kidney disease. Hum Pathol 41: 672–678. 20040395 10.1016/j.humpath.2009.10.008

[54] Gill GV, Baylis PH, Flear CT, Lawson JY (1985) Changes in plasma solutes after food. J R Soc Med 78: 1009–1013. 4067972 10.1177/014107688507801206 PMC1290054

[55] Ciesla DJ, Moore EE, Zallen G, Biffl WL, Silliman CC (2000) Hypertonic saline attenuation of polymorphonuclear neutrophil cytotoxicity: timing is everything. J Trauma 48: 388–395. 10744274 10.1097/00005373-200003000-00004

[56] Pascual JL, Ferri LE, Seely AJ, Campisi G, Chaudhury P, et al (2002) Hypertonic saline resuscitation of hemorrhagic shock diminishes neutrophil rolling and adherence to endothelium and reduces in vivo vascular leakage. Ann Surg 236: 634–642. 12409670 10.1097/00000658-200211000-00014 PMC1422622

[57] Junger WG, Liu FC, Loomis WH, Hoyt DB (1994) Hypertonic saline enhances cellular immune function. Circ Shock 42: 190–196. 8055665

[58] Kolsen-Petersen JA (2004) Immune effect of hypertonic saline: fact or fiction? Acta Anaesthesiol Scand 48: 667–678. 15196097 10.1111/j.1399-6576.2004.00396.x

[59] Shapiro L, Dinarello CA (1995) Osmotic regulation of cytokine synthesis in vitro. Proc Natl Acad Sci U S A 92: 12230–12234. 8618875 10.1073/pnas.92.26.12230 PMC40330

[60] Shao DD, Suresh R, Vakil V, Gomer RH, Pilling D (2008) Pivotal Advance: Th-1 cytokines inhibit, and Th-2 cytokines promote fibrocyte differentiation. Journal of leukocyte biology 83: 1323–1333. 18332234 10.1189/jlb.1107782 PMC2659591

[61] Balgoma D, Astudillo AM, Perez-Chacon G, Montero O, Balboa MA, et al (2010) Markers of monocyte activation revealed by lipidomic profiling of arachidonic acid-containing phospholipids. J Immunol 184: 3857–3865. 20181887 10.4049/jimmunol.0902883

[62] Mantovani A, Allavena P, Vecchi A, Sozzani S (1998) Chemokines and chemokine receptors during activation and deactivation of monocytes and dendritic cells and in amplification of Th1 versus Th2 responses. Int J Clin Lab Res 28: 77–82. 9689547 10.1007/s005990050023

[63] Wang H, Mao Y, Zhang B, Wang T, Li F, et al (2010) Chloride channel ClC-3 promotion of osteogenic differentiation through Runx2. J Cell Biochem 111: 49–58. 20506205 10.1002/jcb.22658

[64] Bikle DD, Ratnam A, Mauro T, Harris J, Pillai S (1996) Changes in calcium responsiveness and handling during keratinocyte differentiation. Potential role of the calcium receptor. J Clin Invest 97: 1085–1093. 8613532 10.1172/JCI118501 PMC507156

[65] Catanozi S, Rocha JC, Passarelli M, Guzzo ML, Alves C, et al (2003) Dietary sodium chloride restriction enhances aortic wall lipid storage and raises plasma lipid concentration in LDL receptor knockout mice. J Lipid Res 44: 727–732. 12562870 10.1194/jlr.M200330-JLR200

[66] Reuter S, Bussemaker E, Hausberg M, Pavenstadt H, Hillebrand U (2009) Effect of excessive salt intake: role of plasma sodium. Curr Hypertens Rep 11: 91–97. 19278597 10.1007/s11906-009-0018-5

[67] Gomer RH, Pilling D, Kauvar LM, Ellsworth S, Ronkainen SD, et al (2009) A serum amyloid P-binding hydrogel speeds healing of partial thickness wounds in pigs. Wound Repair Regen 17: 397–404. 19660048 10.1111/j.1524-475X.2009.00482.x PMC2850269

[68] Pilling D, Vakil V, Gomer RH (2009) Improved serum-free culture conditions for the differentiation of human and murine fibrocytes. J Immunol Methods 351: 62–70. 19818792 10.1016/j.jim.2009.09.011 PMC2783789

